# Learning-Based Approaches to Current Identification from Magnetic Sensors

**DOI:** 10.3390/s23083832

**Published:** 2023-04-08

**Authors:** Sami Barmada, Paolo Di Barba, Alessandro Formisano, Maria Evelina Mognaschi, Mauro Tucci

**Affiliations:** 1 Department of Energy, Systems, Territory and Construction Engineering (DESTEC), University of Pisa, 56122 Pisa, Italy; 2 Department of Electrical, Computer and Biomedical Engineering, University of Pavia, 27100 Pavia, Italy; 3 Department of Engineering, University of Campania “Luigi Vanvitelli”, 81031 Aversa, Italy

**Keywords:** electromagnetic inverse problems, regularization, machine learning, neural networks, measurement uncertainty

## Abstract

Direct measurement of electric currents can be prevented by poor accessibility or prohibitive technical conditions. In such cases, magnetic sensors can be used to measure the field in regions adjacent to the sources, and the measured data then can be used to estimate source currents. Unfortunately, this is classified as an Electromagnetic Inverse Problem (EIP), and data from sensors must be cautiously treated to obtain meaningful current measurements. The usual approach requires using suited regularization schemes. On the other hand, behavioral approaches are recently spreading for this class of problems. The reconstructed model is not obliged to follow the physics equations, and this implies approximations which must be accurately controlled, especially if aiming to reconstruct an inverse model from examples. In this paper, a systematic study of the role of different learning parameters (or rules) on the (re-)construction of an EIP model is proposed, in comparison with more assessed regularization techniques. Attention is particularly devoted to linear EIPs, and in this class, a benchmark problem is used to illustrate in practice the results. It is shown that, by applying classical regularization methods and analogous correcting actions in behavioral models, similar results can be obtained. Both classical methodologies and neural approaches are described and compared in the paper.

## 1. Introduction

Current measurement in aerial power lines, in winding packs for high-field magnets, or in plasmas for industry applications cannot be achieved easily using standard sensors, due to poor accessibility of conductors (e.g., for aerial lines) or to demanding technical issues (e.g., in high field magnets supply), or to harsh environment (e.g., in high temperature plasmas). As a matter of fact, in the proposed examples, not only the total current amplitude but also frequently the current distribution inside the support region (the different conductors in the aerial lines and in high field magnets or the plasma column itself in the latter case) is required. In such cases, the concept of measurement must be understood in a broader sense, and suitable current distribution sensors should be introduced as a combination of magnetic measurements and suited mathematical treatment to cope with the inverse problems of reconstructing current data from magnetic field sensors. The general purpose of the paper is twofold: on the one hand, to provide an overview of effective methods for inverting data from field sensors in order to identify the current distribution and, on the other hand, to test them in a comparative way against a well-known benchmark problem.

The need for these methods is believed to be important because the underlying inverse problem is ill-posed, leading to spurious solutions. The ultimate goal is to pave the way for a virtual sensor system, i.e., a numerical procedure that could help both the current reconstruction, given a set of measurements, and the current source synthesis, given a set of specification on the field distribution in a region of interest.

Recently, many research areas have taken advantage of the potential offered by behavioral models based on machine learning (ML) or (deep) neural networks (DNN) [1,2]. In fact, recent works on the DNN-assisted analysis of electromagnetic (EM) field computation problems showed the promising potential of convolutional neural networks (CNN) and ML tools [3,4,5,6,7,8,9,10,11]. A comprehensive review of recent works on ML for the design optimization of electromagnetic devices can be found in [4], where the growing interest of the community is clearly evidenced. Some works adopted ML or DNN models to predict the key performance indicators of electrical machines [5,6,7], whilst others focused on topology optimization [8,9,10,11].

The main appeal of ML or DNN in dealing with inverse problems is their capability of achieving efficient solutions from experiential knowledge rather than mathematical formulations. On the other hand, such models do not always grant accuracy. A combined use with more classical approaches can be pursued to improve overall performance.

Note that the data used to train the ML or DNN models are inherently bidirectional, and the role of inputs and outputs can be, up to a certain level, interchanged, training the model to directly identify materials, geometries, or sources from measurements of electromagnetic fields. This approach would allow the resolution of inverse problems in much shorter times than by using classical methods, especially when endowed with iterative schemes.

To ease reading, it is fruitful to provide here a definition of inverse problems in terms of the reconstruction of system characteristics, e.g., its inner structure, from observed or desired data. These problems appear in various applications, such as medical imaging with X-rays [12] or other electromagnetic sources [13]. Image processing is the best-known application of behavioral approaches to inverse problems. To cite just a few examples, classical DNNs are compared in [14] with classical sparse reconstruction algorithms, while several CNNs are presented in [15] for medical applications of magnetic resonance imaging. Other possible approaches include recurrent neural networks (where node-connecting weights form a directed graph) and generative adversarial networks (two networks competing into a sort of game [13] each to achieve a different objective in the data processing, regularizing in this way the overall behavior).

In [16], multilayer perceptron (MLP) autoencoders are added to the previously listed approaches. Quite notably, early attempts to solve inverse problems using fully connected neural networks (FCNNs) are reported as early as 1992 [17]. Finally, [18] presents a taxonomy of inverse problems depending on the type of supervision and knowledge of the corresponding direct problem.

Although numerous works introduce ML approaches and NN to solve direct problems in electromagnetism, contributions addressing the inverse case are still rare, yet steadily increasing. Since the inverse problems we are dealing with are classically formulated as the minimization of a reconstruction error, a regularization of the problem (as raw observed data are frequently compatible with multiple solutions) is needed. Usually, to achieve the minimum error, iterative processes are used. While DNN can provide a solution in a single step, when properly trained, much care must be given to the way behavioral approaches regularize the problem. As a matter of fact, the DNN proposes the solution most closely corresponding to the observed data among those considered in the training step. Consequently, DNN does provide an inherent regularization, ruled by the construction of the learning set and by the teaching algorithm: this point needs further investigation in the viewpoint of authors.

In this paper, we first identify the characteristics of various Electromagnetic Inverse Problems (EIPs) usually found in practical cases. Then, we investigate different possible ML and NN approaches to the resolution of the EIP, with particular reference to the bi-directionality of the approach, i.e., to the possibility of training the model by changing the role of input and output of the direct problems, obtaining a straightforward resolution of the inverse problem.

In the first section, we provide a synthetic description of the direct problem we use in the paper and introduce the relevant inverse problem and its mathematical characteristics. The considered EIP is a current synthesis problem: examples of this class being the reconstruction of current distributions from external magnetic measurements, as cited above, or the optimal choice of currents to generate a given field distribution. In the following section, we briefly discuss the classical regularization methods used to allow the resolution of EIPs. Then, we present a short review of available behavioral approaches, together with the numerical techniques used to improve their performance. Finally, we test the proposed schemes on the benchmark problem, which, albeit simple in its scheme, does show all the problems usually faced in more complex cases. To the best of our knowledge, this is the first attempt to assess the inherent regularization capabilities of ML and NN approaches to EIP and to make a comparison between the characteristics of such models and the more classical regularization strategies usually adopted in the resolution of EIP.

## 2. Materials and Methods

### 2.1. Direct and Inverse Electromagnetic (Source) Problems

**Figure 1:**
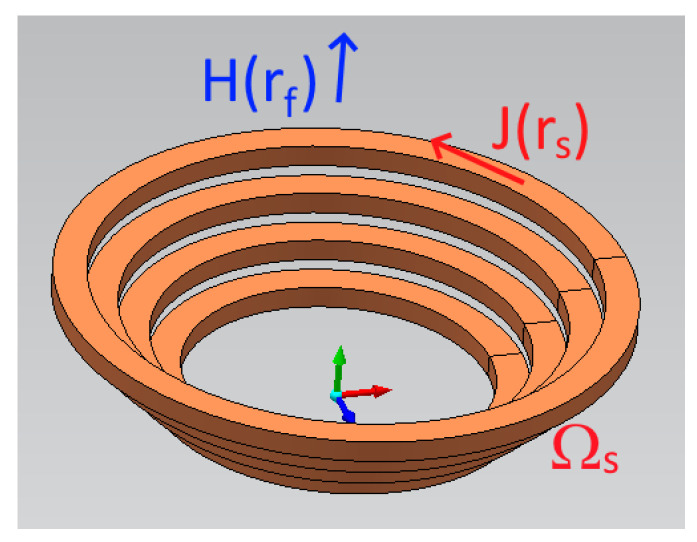
A schematic layout of the source domain Ω_s_ and of the (simulated) field sensors (an example being represented by the blue vector **H**(**r**_f_)).

As described above, data-based models require massive amounts of data in the training step. In the class of problems considered here, such data are related to the measurements of magnetic fields and their sources. To focus on the background theoretical aspects of this problem, we preferred in this paper to use numerically simulated data. In particular, we adopted as a simple example the computation of the magnetic field **H** in free space generated by a set of currents **J** flowing in conductors with known geometry Ω_s_ (Figure 1). Without any pretense of generality, we provide in this section a synthetic description of the mathematical formulation we adopt in the paper for the computation of the magnetic field.

In a homogenous domain, the equation governing the link between current distribution and magnetic field is given by the Biot–Savart integral:

(1) $$H\left(r_{f}\right)=\frac{1}{4\pi }\int_{\Omega_{s}}^{}\frac{J\left(r_{s}\right)\times \left(r_{f}-r_{s}\right)}{{\left|r_{f}-r_{s}\right|}^{3}}d\Omega_{s}$$

where **H** is the magnetic field, **r**_f_ is the position vector of the field points (the sensors), **r**_s_ ∈ Ω_s_ is the source point considered in the integration process, **J** is the source current density, assumed known in the direct problem, and Ω_s_ is the source region. We assume that the field is to be computed externally to Ω_s_, to avoid convergence issues. When **J** is assigned and **H** is unknown, a direct problem arises.

Specifically, to formulate the direct problem in a concise yet explicative form, we can write:

(2) $$H=H\left(J;{\underline{r}}_{f},\Omega_{s};\Omega_{mat};\Omega_{cnd}\right)$$

where **ℋ** is usually an integral operator, such as (1). In (2), **J** represents the input data, while **H** represents the output. The dependence on the current density map **J**, the field point(s) ${\underline{r}}_{f}$ (the underbar sign “_” indicating an array of points), and the source volume Ω_s_ have been highlighted. The formulation (2) is general enough to allow the presence of magnetic or conducting material regions, indicated by Ω_mat_ and Ω_cnd_, respectively, but neglected in this analysis for the sake of simplicity; in addition, we will assume that all materials behave linearly with respect to the current–field relationships. The current material support is known and fixed, and the current is constant, thus allowing a magnetostatic formulation.

Let us now flip our point of view and attempt to formulate the problem of looking for an unknown current **J** from a given field map **H** outside the source regions, which is known as the inverse (source) problem. In this case, we introduce the inverse operator:

(3) $$J=H^{-1}\left(H;{\underline{r}}_{f},\Omega_{s}\right)$$

Equation (3) describes a first type (inhomogeneous) Fredholm equation, generally expressed as:

(4) $$g=\int_{\Omega_{s}}^{}Kf\;d\Omega_{s}$$

where g represents the data of the problem, *f* is the unknown, and **K** is called the kernel of the equation. The possibility of solving the inverse source problem depends on **K**, on the data space (from now on, named Y), and on the solutions space (named X from now on). Let us recall here that the inverse operator **ℋ**^−1^ exists if and only if **ℋ** is bijective, that is:

(5) $$\forall H\in H(X)\subset Y,\;\exists !\;J\in X|H^{-1}(J)=H$$

and

(6) H(X)=Y

The set H(X) is known as the rank of the operator. Unfortunately, the plain existence of an operator **ℋ**^−1^ is not enough for **ℋ** to be invertible, since the solution can be not unique. It is possible to state that a linear operator **ℋ** is invertible if it has a bounded inverse.

We will use the additional statement that a compact linear operator **ℋ** admits a bounded inverse **ℋ**^−1^ if its rank, H(X), has a finite dimension. Thus, as a conclusion, we can state that, to have a stable solution, we need **ℋ**^−1^ to be a linear, compact operator, and its rank (**ℋ**^−1^(Y)) to be of finite dimension. Unicity also requires X to be of finite dimension.

Our operator **ℋ** is an integral operator, defined by a kernel **K**, which, in the case we are considering, is the fundamental solution of the magnetostatic problem as described in (1). This implies:

(a) **ℋ** is a linear operator;
(b) the kernel function is well-behaved (smooth, continuous, etc.), quadratically bounded, and grants compactness to **ℋ**. So, according to what was stated before, we only need to constrain the data and solution spaces to have a finite dimension.

We usually have a discrete set of measurements, and we need to elaborate them numerically. In order to distinguish the theoretical field **H** generated by **J** from the one actually measured, which is affected by uncertainties and noise and generally known in a (discrete) subset of points, we will indicate the array of available field measurements as M**_H_**. In addition, from a practical point of view, current distribution can usually be represented by a set of parameters, the most straightforward being the current amplitude in the diverse conductors, but a different view can be the coefficients of current density map in some representation bases. In any case, we will use the symbol I to indicate the solution parameters array. Under the assumed linearity hypothesis, the discrete nature of both sources and measurements allows one to postulate the existence of a matrix transforming the former into the latter. This matrix is usually called the lead field matrix, and we will be indicating it by the symbol $\underline{\underline{A}}$. In the example problem we are considering, the elements a_mn_ of $\underline{\underline{A}}$ can be computed by evaluating the Biot–Savart integral (1) on the n-th conductor Ω_n_ in the m-th field point **r**_m_.

Having achieved discrete finite-dimensional data space Y and solutions space X, we have demonstrated that the problem admits a unique solution, but this does not yet grant well-posedness since the solution may depend not smoothly on data. If this is the case (as it usually is), we must be aware of the impact of noise and approximation and select the best discrete approach. We must, in any case, keep in mind that a problem in the resolution process usually is not a consequence of lack of data, but rather a consequence of the nature of the operator or a consequence of a wrong choice of data and solution spaces.

### 2.2. Regularization Methods: A Review

The correct approach to obtain solution uniqueness is to adopt regularization methods. In this section, we present a comparative review of some among the best-known regularization methods and their application to the proposed current identification problem. We consider the following schemes:

1. Classical (direct) methods: Tikhonov method, Truncated SVD, and ν-Method;
2. Statistical methods: Linear Regression, linear fit with Principal Component Analysis, and Elastic Net Regularization.

The classical and statistical methods considered here are based on the properties of the lead field matrix $\underline{\underline{A}}$, computed using the Finite Element Method (FEM). Both classes of methods apply as well in the case of lead field matrix recovered from a purposedly designed set of measurements.

#### 2.2.1. Direct Methods

The classical linear inverse problem I = $\underline{\underline{A}}$^−1^M_H_ (where $\underline{\underline{A}}$^−1^ must be understood as the Moore–Penrose pseudo-inverse) has been tackled using many different approaches for its regularization. A non-exhaustive list may include the Tikhonov approach (TA, [19]), the Truncated Singular Value Decomposition (T-SVD, [19]), and the Discrepancy Principle (DP, [20]). A new group of methods, collectively known as iteration-based, has started to be considered more recently. Examples are the ν-Method (νM, [21]) and the ART method [22]. A broader list of possible regularization schemes can be found in [23,24]. We just briefly describe here those that are considered in the following for the comparison with the behavioral models.

- TA: The Tikhonov approach is probably the most diffused counter measure to the ill-posed nature of inverse problems. In the notation adopted here, the solution process of the (regularized) inverse problem can be cast as:
  (7) $$\underset{\underline{I}}{\min}\left({\left\|\underline{\underline{A}}\underline{I}-{\underline{M}}_{H}\right\|}_{Y}+\lambda {\left\|\underline{I}\right\|}_{X}\right)$$
  where ${\left\|\cdot \right\|}_{Y}$ represents the 2-norm of the measurements vector, ${\left\|\cdot \right\|}_{X}$ represents the 2-norm of the parameters vector, and *λ* is the regularization parameter. The performance of the TA depends on the parameter *λ*, balancing the model error and the solution norm. The L-curve approach [25], or alternatively the generalized cross validation method [26], are the most adopted strategies to choose its value.
- T-SVD: The Truncated Singular Value Decomposition is based on the representation of $\underline{\underline{A}}$ in terms of its left and right singular vectors:
  (8) $$\underline{\underline{A}}=\sum_{i=1}^{N}s_{i}{\underline{u}}_{i}{\underline{v}}_{i}^{T}$$
  where *u*_i_ and *v_i_* are orthonormal vectors in the currents space and in the measurements space, respectively; *s_i_* are the singular values of $\underline{\underline{A}}$, in descending order; and N is the matrix rank. To obtain a (rank-deficient) well-conditioned matrix $\underline{\underline{A}}$_n_, it is possible to truncate the summation to an index *n* < N. The pseudo-inverse $\underline{\underline{A}}$^−1^_n_ provides a (regularized) solution: I_n_ = $\underline{\underline{A}}$^−1^_n_ M_H_. The smaller is n, the smoother but less detailed will be the solution.
- νM: It can be shown that iterative algorithms (e.g., conjugate gradient) allow smoother components of the solution of the linear problem $\underline{\underline{A}}\underline{I}-{\underline{M}}_{H}$ to converge earlier. The ν-Method leverages this property to regularize the resolution process by stopping the iterations before complete convergence. The role of a regularizing parameter in this case is played by the number of iterations.

#### 2.2.2. Statistical Approaches

Statistical approaches can be used to solve inverse problems when a dataset of correlated source and measurement values is available. Taking inspiration from experimental physics, we can extract some relationship (e.g., a linear interpolation) between the outputs, in our case, magnetic fields, and the inputs, in our case, the currents, fitting the model to the data. Under suitable hypothesis on the distribution of the data and on the underlying actual model, as presented in Section 2, the fitted model will be able to provide reliable estimates of the output as well as for unseen inputs. Note that also in the case of fitted models, the ill-conditioned nature of the underlying problem amplifies data nuisances, and some regularizing techniques should be applied. We will briefly analyze here a few well-known interpolation approaches.

- MLR: Multi-Linear Regression adopts linear regression model from multiple data m_k_ (k = 1, 2, …, N_meas_) to multiple output I_i_, expressed by:
  (9) $$I_{i}=\beta_{i0}+\sum_{k=1\ldots N_{\mathrm{meas}}}^{}\beta_{ik}m_{k}+\varepsilon_{i}\;i=1,2,\ldots N_{\mathrm{curr}}$$
  where βi_0_ and β_ik_ k = 1 … N_meas_ are the interpolation coefficients, and ε_i_ is the residual error, due to additive white Gaussian measurement noise, for example. Currents I_i_ are fitted independently. Least squares minimization is used to estimate the fit coefficients. Thanks to the assumptions on the noise, the coefficients also maximize the likelihood of the prediction vector.
- LPCA: Linear fit with Principal Component Analysis starts from the assumption that the information about the (required) field map is highly redundant, so any regression model should probably address such an issue. This is easily verified from the lead field matrix analysis and from the correlation analysis of the field measurement. In such cases, PCA can be used to extract the most effective regressors. This helps in regularizing the problem, as PCA removes any redundancy among input data. The elements of the orthogonal basis made of principal components can be ranked in a decreasing order of variance over the data set, and reduced models explaining any desired level of data variance can be obtained.
- ENR: Elastic Net Regularization is a regularization technique minimizing regression coefficients of the less relevant variables. For each reconstructed variable (currents, in our example), the ENR technique solves the following minimization problem to find the set of interpolation coefficients β_0_, β_k_, k = 1 … N_meas_ [27]:
  (10) $$\left\{\beta_{0},\underline{\beta }\right\}=\left(\frac{1}{2N_{Samples}}\sum_{l=1,N_{Samples}}^{}{\left(I_{l}-\beta_{0}-{\underline{M}}_{l}^{T}\underline{\beta }\right)}^{2}+\lambda P_{\alpha }\left(\underline{\beta }\right)\right)$$
  where α∈]0,1[, *λ* is a nonnegative real number, and $P_{\alpha }\left(\underline{\beta }\right)=\frac{\left(1-\alpha \right)}{2}{\left\|\underline{\beta }\right\|}_{2}^{2}+\alpha {\left\|\underline{\beta }\right\|}_{1}$. Note that ENR for *α* = 1 reduces to lasso regularization, while for *α* → 0, it approaches ridge regression.

### 2.3. Machine Learning and Neural Network Models for Electromagnetic Inverse Problems

The data-driven statistical approaches described in Section 2.2.1, i.e., learning a behavioral model using an available collection of paired input–output quantities, is the basic operating principle of supervised learning algorithms such as NN and other ML algorithms. The use of ML is a natural choice when the behavior of the model is generally too complex to be efficiently described analytically, or perfect knowledge of physical parameters is lacking, and this is the case in many inverse and direct problems involved in electromagnetic applications.

The success of NN and other algorithms, such as support vector machines, is due mainly to two factors: they are universal approximators, and their generalization and regularization capabilities can be controlled in several different ways. For example, regularization can be improved by diminishing the number of neurons in the hidden layer by early stopping of the training (which is equivalent to νM or ART regularization techniques), by using a regularization term in the loss function that penalizes the presence of large neural weights (which is in a sense similar to the TA), or by the so-called dropout method that randomly removes a certain number of neural connections.

A further, relevant consideration exists regarding the dimensionality of the input and output vectors. In fact, when the number of outputs exceeds the number of inputs, we are asking the model to generate redundant information possibly not present in the input itself, and this usually leads to poor performance of training algorithms. When using classical approaches, standard countermeasures include the adoption of a regularization technique. In the case of ML or NN, other possibilities are available. As a matter of fact, it would be preferable to apply a dimensionality reduction technique to the output data before training the model (either ML or NN) or to add some a priori information, as in the case of Physics-Informed Networks [28].

On the other hand, when the number of inputs is greater than the number of outputs, NNs perform quite satisfactorily. However, if the number of inputs is very high or there are many linearly dependent inputs, the neural model can be affected by the course of dimensionality. In this case, a dimensionality reduction of the inputs is again recommended. As a result, in many cases, it is necessary to exploit the methods for reducing dimensionality, which can be linear, such as PCA, or nonlinear, such as autoencoders [1]. Some models, such as DNN, can deal directly with high dimensional inputs, avoiding the need to reduce the number of features. In any case, a preliminary PCA is usually very helpful and adds valuable knowledge, revealing the directions along which data points are most distributed and how much information is lost when cutting negligible directions. In addition, in many cases, PCA is strongly related to mathematical features of the inputs data, which can be directly linked to a physical behavior of the system.

In the remainder of the paper, the authors use a benchmark problem, described in Section 3, to test different data-driven approaches to solve the EIP. Three different EIP solution procedures are implemented and briefly described as follows:

#### 2.3.1. EIP Using Neural Networks and Deep Learning

**Figure 2:**
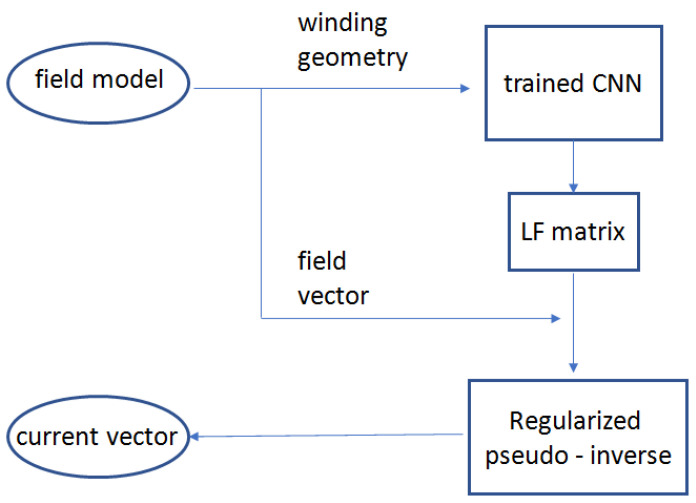
Block diagram of the method based on Neural Networks and Deep Learning.

In this contribution, the forward operators consist of a dataset of Finite Element Models (FEMs) generating lead field matrices $\underline{\underline{A}}$ for different choices of the geometrical quantities. Then, a PCA is applied to represent the $\underline{\underline{A}}$ matrices in a lower-dimension feature space so that the original matrix can be well reconstructed from a reduced set of principal components. Then, we train an NN to predict the reduced set of principal components given the geometry of the system. The corresponding Lead Field (LF) full matrix is then reconstructed from the predicted principal components. Subsequently, the EIP can be solved by means of one of the above-mentioned regularization methods. In particular, the pseudo-inverse is computed by means the T-SVD approach. A scheme of this method is shown in Figure 2.

The proposed method, which combines a CNN with an inversion technique, is very general because it is geometry-free. In fact, the network learns the equations, so it is able to generalize to new geometries, enabling a rapid solution of the synthesis problem.

With reference to this approach, a further remark can be made. It is common practice in many applications to solve an EIP by implementing an optimization procedure: the direct problem (from known sources and geometry to the measured field values) is solved iteratively, allowing the calculation of a fitness function; sources (and geometry) are updated to reach the desired value of the fitness function. At the end of the procedure, the resulting model is solicited with a set of (desired) field measurements, and a set of sources are obtained. Mathematically, we first obtain a model $H_{M}^{}$ of the forward operator **ℋ**, and the optimization algorithm searches for the best currents I, minimizing an objective function of the form ${\left\|H_{M}\left(\underline{I}\right)-{\underline{M}}_{H}\right\|}^{2}$.

This implicit approach may find accurate results, but it is computationally expensive; for instance, in electromagnetics, $H_{M}\left(\underline{I}\right)$ is often evaluated by a numerical procedure (i.e., Finite Element Method, Boundary Element Methods, Integral Methods, etc.). The optimization phase is not relevant for the present comparative study, and it will be addressed in future works. However, ML can also play a fundamental role in this case; in particular, the ML-based surrogate model of **ℋ** that can be obtained with the NN-LF approach can be used to solve the direct problem at each iteration, resulting in a dramatic reduction of the overall computational time.

Alternatively, without an explicit use of the lead field matrix, a direct estimation $H_{ML}^{-1}$ of the inverse operator $H_{}^{-1}$ is obtained using different ML paradigms. In particular, one implementation of $H_{ML}^{-1}$ is obtained by training shallow neural networks with sigmoidal activation function, using different learning approaches. A second ML approach is considered, and a deep neural network, i.e., composed of multiple layers, is trained and tested.

Learning the inverse operator by training an NN allows us to exploit the different and powerful regularization approaches usually adopted in NN training steps, such as early stopping with a validation set or Bayesian regularization. Moreover, the shallow fully connected sigmoidal NN being a universal approximator, it is likely to correctly learn and represent the inverse model from the training data. A deep, multi-layer neural network is an alternative approach that is often heuristically found to outperform the shallow neural network, also allowing us to use a combination of linear and nonlinear layers in order to take into account previous knowledge on the model that generated the dataset.

#### 2.3.2. EIP by Linear Regression

This approach consists of learning an estimate $H_{L}^{-1}$ of the inverse operator $H_{}^{-1}$ from the data to predict the currents I from the magnetic measurements M_H_. We denote this approach as the explicit inverse model. The main advantage of this approach is that, once the model is trained, it can perform the inversion in an extremely short time. Of course, a training dataset containing N_samples_ observations is necessary. One disadvantage is that if the geometry of the system changes, the model is no longer valid: a new training should be performed on a newly generated dataset. In particular, the use of Standard Regression Algorithm (SRA), Robust Regression Approaches (RRA), and Truncated Singular Value Decomposition (T-SVD) pseudo-inverse is investigated and applied to the benchmark problem.

## 3. The Benchmark Problem

In this section, the benchmark problem used to numerically evaluate the performances of the different approaches mentioned above is described [29,30].

### 3.1. The Forward Problem

**Figure 3:**
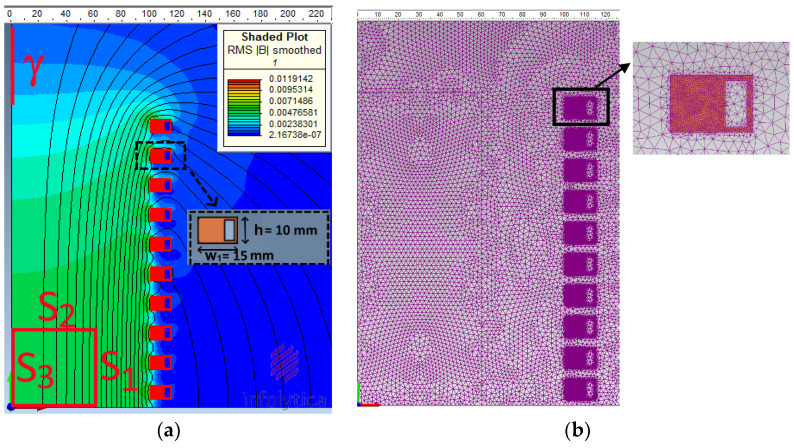
Geometry, controlled regions, and magnetic flux lines (**a**); discretized domain (**b**).

A multi-turn air-cored winding is considered (Figure 3). The winding, which is composed of 2N_curr_ independent turns, is suitable for in vitro experiments of magneto-fluid hyperthermia. The geometry is axially symmetric, so a Poloidal (r, θ, z) frame is adopted, and θ variations are considered negligible (see Figure 1 for a 3D sketch of the geometry). The direct problem, i.e., computing the magnetic field **H** (or, in our benchmark, the flux density **B**), given the DC source currents I and the coil geometry, is defined as an axisymmetric system in static conditions. No polarizable magnetic materials are present, and the problem can be considered linear. Notwithstanding the absence of ferromagnetic materials, due to the hollow shape of the conductors, the forward field analysis is better approached by the FEM method.

### 3.2. The Current Identification (or Synthesis) Problem

The aim of the problem is to select the N_curr_ currents to generate a uniform flux density map with a prescribed value **B**_0_, uniform within a region adjacent to the symmetry plane z = 0 inside the coils and with an amplitude as small as possible outside the winding. In such a situation, it is reasonable to limit the search for the best current distribution to configurations that are symmetric with respect to the symmetry plane, thus reducing the unknown currents to just N_curr_ = 10. In order to evaluate the field uniformity in the inner region of interest (ROI), the magnitude of the flux density field **B** is sampled over 30 field points, evenly spaced on the boundary of the ROI (lines S_1_, S_2_, and S_3_ in Figure 3); moreover, the field is sampled on 10 points along the outer line γ. All 40 sensors are considered to be point-wise and ideal probes. Axial flux density components are measured only on S_3_ and γ, so the total number of considered measurements is N_meas_ = 60. More details on the benchmark problem geometry can be found in [29].

Starting from this background, the current identification problem is defined as:

*Find the current distribution I that minimizes the discrepancy between the trial flux density **B**(r,z) and the measured flux density **B**_0_(r,z) at the sensors location*

where **B**_0_ = (B_r0_, B_z0_); B_r0_ = 0 and B_z0_ = *K* along S_1_ and S_2_; B_z0_ = *K* along S_3_; B_z0_ = 0 along γ; *K* represents the desired field level (2.00 mT is assumed in the present work), and advantage is taken from symmetry being B_r0_ = 0 on the axis *r* = 0.

#### Dataset Generation

**Figure 4:**
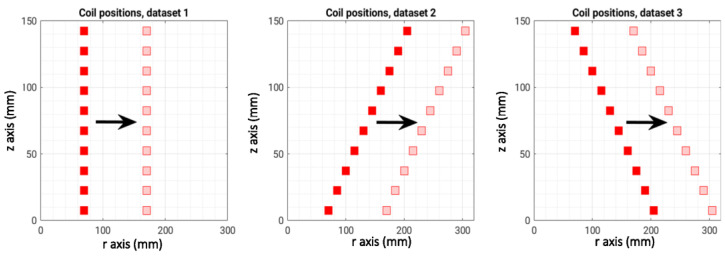
Coil positions in the three datasets.

Figure 4 shows the geometrical details of the three datasets: in the first one, the 10 coils have the same radius (cylinder), in the second one, the radius linearly increases with increasing height (diverging cylinder), and in the last one, the radius linearly decreases with increasing height (converging cylinder). Each subfigure of Figure 3 shows the first and the last cylinders of the dataset; the other cases are characterized by intermediate values of the x-coordinate of each conductor. For each dataset, N_samples_ = 401 different cases (also called instances) were generated, and, for each instance, the corresponding lead field matrix was saved. Consequently, we have a collection of lead field matrices $\underline{\underline{A}}$ of dimension $N_{cur}\times N_{meas}=10\times 60$.

## 4. Results

### 4.1. Case Studies

Given the datasets described in the previous section, generated by the simulation of the benchmark (linear) problem, the different approaches recalled in Section 2 are here developed and discussed in detail.

#### 4.1.1. EIP by the Approximation of the Lead Field Matrix

The aim is to obtain a surrogate model of the lead field matrix, and for the reasons mentioned in previous sections, we first try to investigate the possibility of representing the $\underline{\underline{A}}$ matrices with a lower number of features using PCA.

Firstly, we reshape the 60 × 10 matrices to a one-dimensional vector with 600 values by row stacking. All the collected matrices are then represented by a dataset matrix of dimension 401 × 600, where rows are observations and columns are variables. By applying PCA to such a matrix, we calculate that 99.99% of the total variance can be explained using the first three principal components for all of the three datasets above. A good data reconstruction capability is then expected using a low number of principal components.

**Figure 5:**
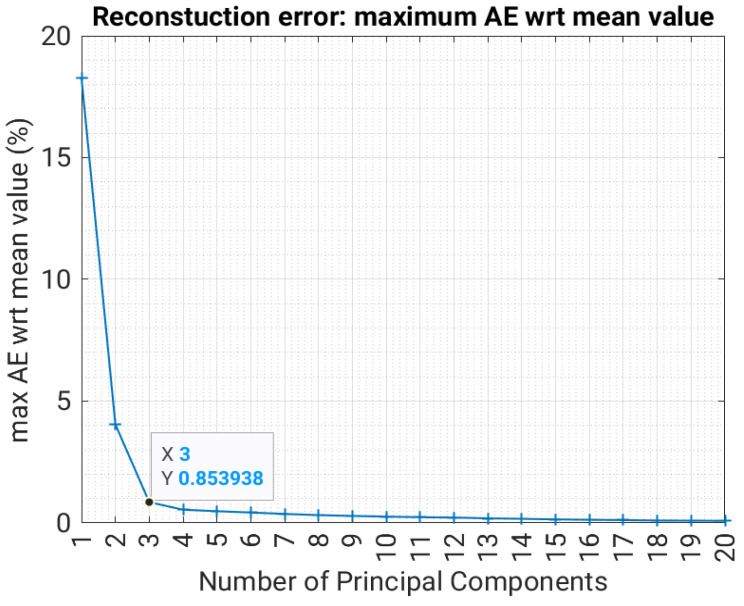
Reconstruction error as a function of the number of principal components for Dataset 1.

To evaluate the reconstruction error as a function of the principal components, we first split the dataset into 80% training and 20% validation (we do not perform the test here, since we are interested just in the characteristics of the learning phase). We learn the PCA representation using the training set only, and we apply the learned PCA projection to the validation data. Then, we use a reduced number of principal components to reconstruct the original $\underline{\underline{A}}$ matrices of the validation set, and we calculate the reconstruction error *AE* as follows:

(11) $$AE=\frac{\langle max\left(\left|\underline{\underline{\hat{A}}}-{\underline{\underline{A}}}_{t}\right|\right)\rangle }{\langle max\left(\left|{\underline{\underline{A}}}_{t}\right|\right)\rangle }\%$$

where $\underline{\hat{\underline{A}}}$ is the predicted matrix, ${\underline{\underline{A}}}_{t}$ is the target matrix, max refers to the elements of each matrix (or difference matrix) in the test set, and the average operator < > acts over the validation data set composed of N_test_ samples of the coil distribution. Figure 5 shows the trend of the reconstruction error *AE* with respect to the number of principal components for Dataset 1. Similar figures are obtained for Datasets 2 and 3 as well.

With three principal components (PCs), the maximum absolute error is below the 0.85% of the mean absolute field value for Datasets 1 and 2, while it is below 0.5% for Dataset 3. At this point, we can efficiently represent the $\underline{\underline{A}}$ matrices in a lower-dimensional space using just three or four principal components.

**Figure 6:**
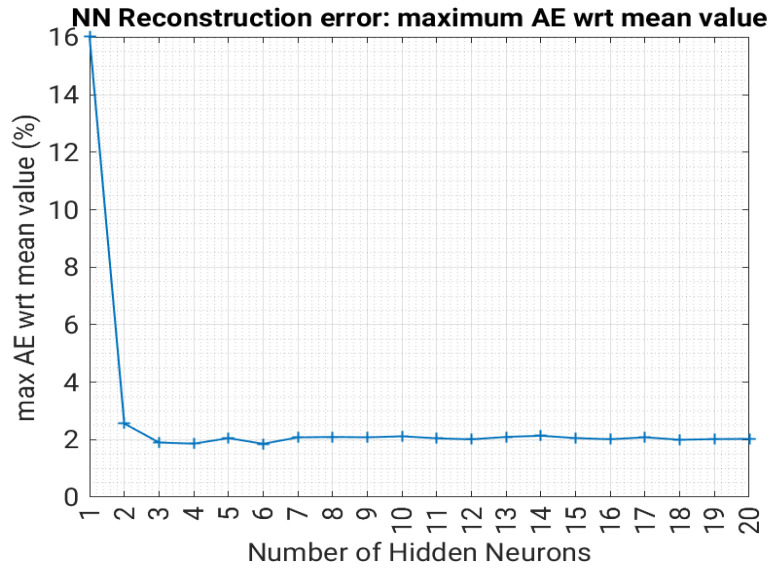
Reconstruction error as a function of the number of neurons for Dataset 3.

A shallow NN with sigmoidal activation functions was trained to predict the first 3 principal components of the lead field matrix $\underline{\underline{A}}$ given as the input vector of the corresponding 10 radii. We used the backpropagation algorithm based on Bayesian Regularization, which allows fast convergence and avoids overfitting [31]. To select the number of neurons in the hidden layer, the trained NN was validated over validation data. In particular, validation errors are averaged over 10 runs with shuffled data. The predicted three principal components values, obtained as the output of the neural network, were used to reconstruct the corresponding $\underline{\underline{A}}$ matrix, and we calculated the reconstruction error as previously defined, obtaining the result shown in Figure 6.

When the number of neurons is greater or equal to three, the reconstructed matrix has a maximum error within the 2.5% of the average value of the target matrix for Datasets 1 and 2, while the error is 2.0% for Dataset 3.

As a final comment, the reconstruction of the lead field matrices (direct problem) did not pose difficult learning tasks either for the statistical approaches using PCA or the NN approaches.

**Figure 7:**
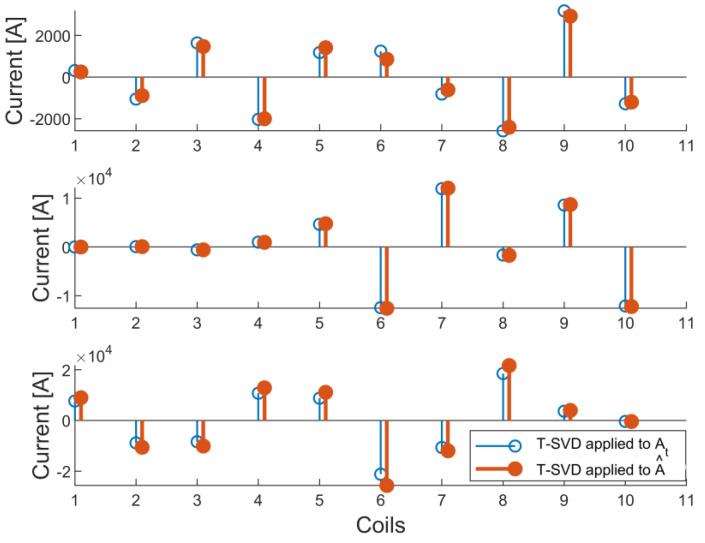
Currents from T-SVD method (8 singular values retained) applied to ${\underline{\underline{A}}}_{t}$ (blue dot) versus currents from $\underline{\hat{\underline{A}}}$ (red dot): cylinder case (Dataset 1): up, diverging cylinder (Dataset 2): middle, converging cylinder (Dataset 3): down.

For solving the inverse problem, the T-SVD method considering the first eight singular values is applied for inverting the lead field matrix $\underline{\underline{A}}$. In particular, a comparison between the currents reconstructed by means of the ${\underline{\underline{A}}}_{t}$ matrix obtained with the FEM and the $\underline{\hat{\underline{A}}}$ matrix predicted by the NN is made in Figure 7 for a selection of test cases.

In Figure 7 and in the other similar figures, we represented the 10 reconstructed currents in a subset of cases from the dataset using dots with different colors. Comparison to true values is also reported when it is useful.

If no regularization method is applied, quite large reconstruction error can be observed. To compare regularized and non-regularized results, we show the average reconstruction errors in Table 1.

#### 4.1.2. EIP Using Linear Regression

Reconstructing directly the inverse matrix $\underline{\underline{A}}$^−1^ requires specific measures to regularize the problem. For the sake of conciseness, the numerical results shown here are only relative to the case with equal radii (Dataset 1). The first tests have been performed using Standard Regression Algorithm (SRA), corresponding to the MLR technique in Section 2. SRA achieved quite poor results, producing a matrix ${\hat{\underline{\underline{A}}}}_{SRA}^{-1}$ with just 10 non-vanishing columns, with a rank equal to 10, coherent with the rank of the true lead field matrix. The thusly obtained matrix ${\hat{\underline{\underline{A}}}}_{SRA}^{-1}$ is capable of interpolating training data, but its generalization capabilities, that is, the capability of providing results (currents from measurements) for examples not included in the data used for regression, are quite poor. Using Robust Regression Approaches (RRA) based on ENR with α = 1 (thus minimizing the norm of the output together with the interpolation error on noiseless data from the magnetic sensors), we obtained a matrix estimate ${\underline{\underline{\hat{A}}}}_{RRA}^{-1}$ providing a good approximation to the Moore–Penrose pseudo-inverse $\underline{\underline{A}}$^−1^. On the other hand, when testing the reconstructed matrices on noisy validation data, poor results are obtained for ${\underline{\underline{\hat{A}}}}_{RRA}^{-1}$, similar to those achieved by $\underline{\underline{A}}$^−^^1^. As a further possibility, we trained a linear model using RRA and noisy data. Generating repeated instances of measurements with Additive White Gaussian Noise (std. deviation equal to 0.1% of full scale) and then averaging, the validation errors (on different subset of noisy examples) matched those obtained using a T-SVD pseudo-inverse ${\hat{A}}_{PINV}^{-1}$, with 6 singular modes (out of the possible 10).

**Figure 8:**
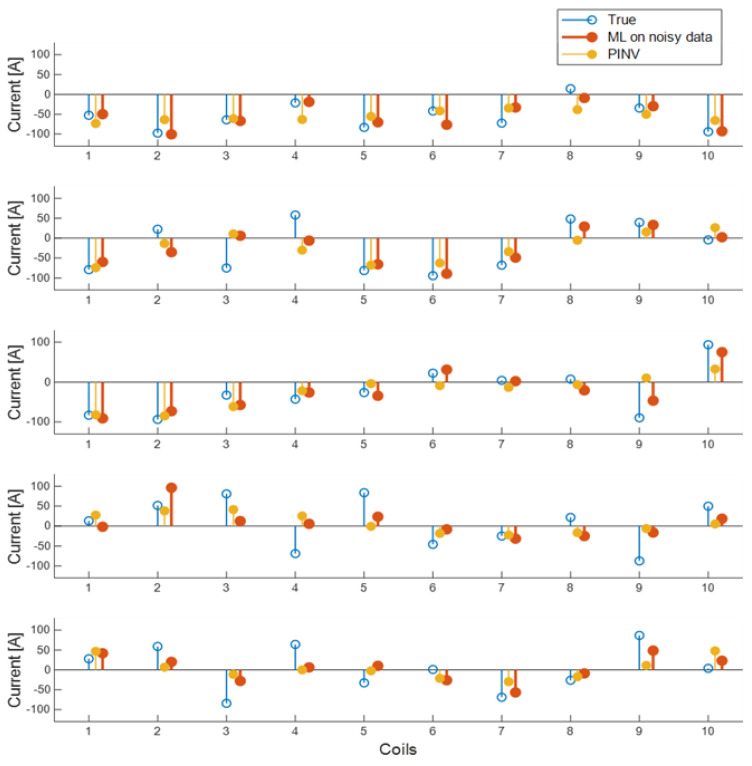
Five examples of reconstructed currents in the validation data set. Blue: true currents; red: reconstructions using RRA with noisy data; and yellow: reconstructions using pseudo-inverse with six singular modes kept.

Results in terms of (inverse) matrix reconstruction error *I*AE are reported in Table 1 for different approaches, where possible. Table 1 also reports a similarly defined current reconstruction error (CE):

(12) $${CE}_{validation}=\frac{{\langle max\left(\left|\underline{\hat{I}}-{\underline{I}}_{t}\right|\right)\rangle }_{validation}}{I_{max}}\%$$

where $\underline{\hat{I}}$ is the predicted currents array, ${\underline{I}}_{t}$ is the target current array, and I_max_ is the maximum allowed current, 100 A in our case. A similar error index *CE_train_* is defined using the training set rather than the validation set in order to assess the generalization capabilities of the different approaches. To keep results comparable, Table 1 reports also the errors in the case of NN-based approaches. Note that in the case of NN, it is not possible to define the matrix reconstruction error, since the networks create their own model. In addition to showing the generalization capability of the RRA on noisy data on actual currents, Figure 8 reports a few examples of reconstructed currents extracted from validation data.

Note that the problem is linear; hence, the relative error of the output is given by the relative error of input times the conditioning number of the matrix. If no regularization method is applied, even a small error in the data produces a large error in the estimates.

**Figure 9:**
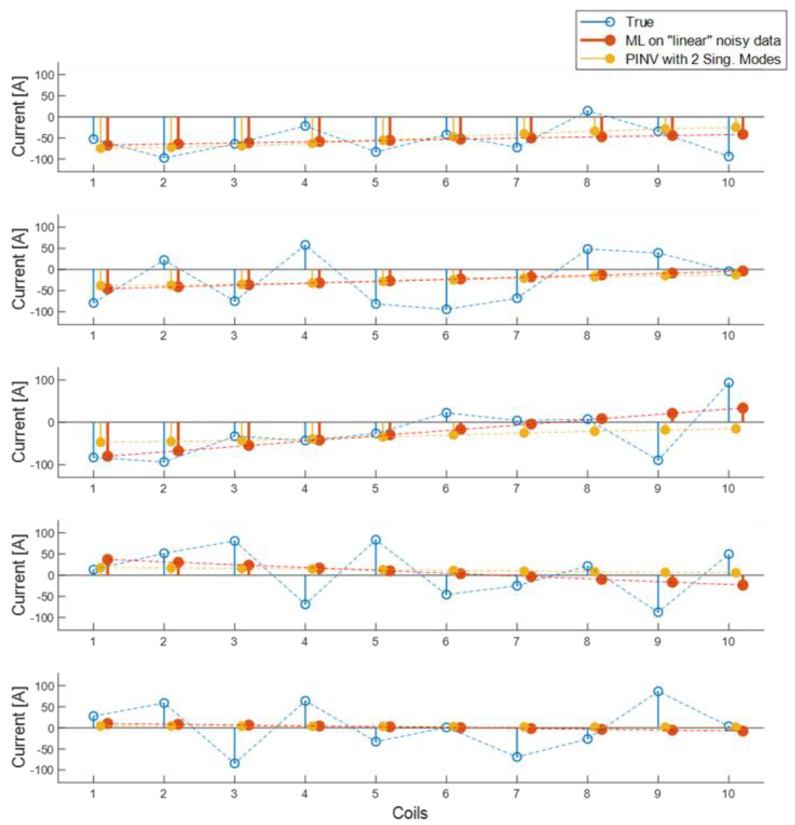
Five examples of reconstructed currents in the validation data set, using only linear current distribution to create a regression model. Blue: true currents; red: reconstructions using RRA with linear noisy data; and yellow: reconstructions using pseudo-inverse with two singular modes kept. Dashed lines connecting the values highlight the trend of the currents with respect to the conductor index.

Quite interesting is the case of training an RRA model using data where only linear current distributions are allowed. In this case, the matrix ${\hat{A}}_{RRA}^{-1}$ of the trained model has just two non-vanishing columns (which complies with the rank in the data matrix). When validating the model on the validation noisy data (with uniformly distributed currents), the forecast currents resemble those obtained using a T-SVD pseudo-inverse with just two singular modes retained (see Figure 9). This result shows how strongly the reconstruction capability depends on the choice of examples in the training set, similar to how the use of T-SVD in classical approaches produces results strongly affected by the choice of truncation level, limiting the number of retained matrix singular vectors.

#### 4.1.3. EIP Using Neural Networks and Deep Learning

The first attempt was carried out using a shallow NN with sigmoidal activation functions. Using ”noiseless” data with the standard Levenberg–Marquardt backpropagation algorithm [32] produces an NN with poor generalization capabilities, similar to standard regression models or to Moore–Penrose pseudo-inverse without truncation (see Table 1). To improve the NN generalization capability, a Bayesian learning approach was tested. The Bayesian approach minimizes a linear combination of squared errors and weights, modifying the linear combination to achieve good generalization capability at the end of the training phase [33]. In addition, multiple shallow NNs were trained on different instances of the same noisy learning data set (with the same AWGN as in the RRA case). Results were then averaged to achieve the final, robust shallow NN model.

**Figure 10:**
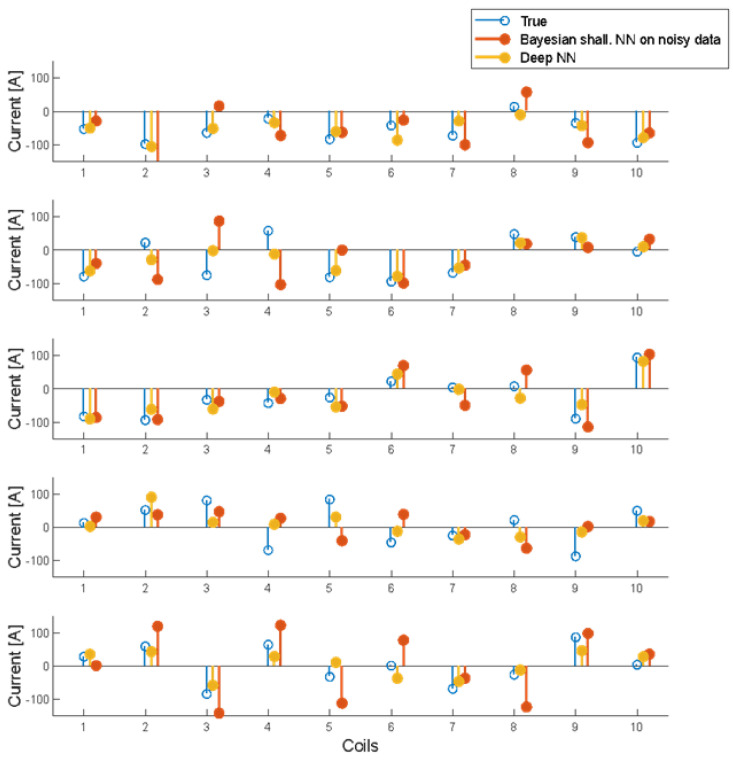
Five examples of reconstructed currents in the validation data set. Blue: true currents; red: reconstructions using a shallow NN trained with Bayesian rule, on noisy data; and yellow: reconstructions using a DNN with a first linear layer to encode data (60 inputs, 10 hidden neurons) and a second sigmoidal layer (from 10 hidden to 10 output neurons).

The high correlation among input data (measurements) suggests using a Deep NN, i.e., a multilayer network with a first layer made of linear neurons, to ”learn” a data compression rule. Using 10 neurons in the first layer forces the DNN to look for the most effective 10 linear combinations of measurements, achieving good results already with 10 neurons in the second, sigmoidal layer. This approach reduces the number of weights to train, and the process is faster. Results are reported in Table 1 and in Figure 10 on a few examples of the validation set to better show the actual quality of reconstructed currents.

## 5. Discussion and Conclusions

The problem faced here, although showing a simple structure to ease comprehension and reduce computational burden, shows all the pitfalls of electromagnetic inverse problems. In our opinion, the difficulties in the resolution of the problem using classical approaches are intrinsic in their mathematical structures, as discussed in Section 2, and cannot be overcome by a plain, straightforward application of machine learning. This point has been demonstrated, in our opinion, by the poor performance of simple, non-regularized regression or neural approaches. On the other hand, regularization schemes are available also for the latter, so we compared regularized neural networks with similar classical schemes, showing how NNs have the capability of extracting the underlaying relationships quite naturally, with minimal tailoring of learning schemes. This is not always the case for classical approach, a typical example being the choice of the truncation threshold required in the T-SVD approach or the choice of the regularization parameter in the Tikhonov regularization.

In our opinion, many similarities can be found between some classical regularizations and the way neural networks need to be trained to achieve satisfactory results. As an example, the νM classical approach aims to prevent the overfitting of the dataset, using the jargon of neural network practitioners. As a second example, the Bayesian learning aims to minimize the weights of the network, thus bearing some similarities with the Tikhonov regularization. Conversely, the intrinsic feature of repeated examples presentation, eventually in varying order, gives to the training process of neural networks an effective capability of dealing with noisy, imprecise data, which is not a characteristic of any classical algorithm, although it can be transferred to regression algorithms by dividing the data set into smaller sets and fitting repeatedly on each of them. Note that while the handling of ill-posedness in the classical approaches has been designed specifically for the resolution of inverse problems and benefits from long-lasting experience for parameter tuning, the countermeasures adopted to improve NN performance are rather general purpose ones, and we are convinced that better results could be achieved by fine tuning their parameters.

As a conclusion, the adoption of machine learning and, more specifically, neural networks, provides new tools for the resolution of (electromagnetic) inverse problems. The underlaying ill-posed nature of these problems, nevertheless, must also be dealt with when adopting data-based approaches. The main contribution of this paper, using a simple yet illustrative benchmark problem, is the attempt to compare some of the classical well known regularization schemes with some measures adopted in the training of machine learning or neural model.

In our opinion, there are correspondences between many classical regularization approaches and countermeasures used to allow NN to converge. A few were highlighted in this paper, but we are convinced that many others can be found.

## Figures and Tables

**Table 1:** Reconstruction errors for different approaches.

	$\underline{\underline{A}}$^−1^ Moore–Penrose Pseudo-Inverse	T-SVD Threshold 10^−7^	T-SVD Threshold 10^−4^	Standard Regression (SRA)	Robust Regression (RRA on Noisy Data)	Shallow NN	Shallow NN with Bayesian Training	Deep NN with Linear Layer	Deep NN with Linear Layer on Noisy Data
*I*AE	-	99.3%	100.0%	320.0%	99.7%	-	-		-
*CE_test_*	2065.0%	63.1%	91.8%	4165.5%	59.2%	7169.8%	145.4%	612.4%	64.1%
*CE_train_*	0.0%	53.6%	91.8%	0.0%	59.2%	0.0%	87.4%	28.3%	56.8%

## Data Availability

The data presented in this study are available on request from the corresponding author. The data are not publicly available due to the extremely large overall size.
